# Body and Mind at Cambridge

**Published:** 1891-03-07

**Authors:** 


### The Hospital

A Weekly Journal of

**Science, Medicine, Nursing, and Philanthropy.**

*For Hospitals, Asylums, Infirmaries, Dispensaries, Medical Practitioners, Students, Nurses, and the Charitable Public.*

Vol. IX.—No. 232.] [March 7, 1891.

### BODY AND MIND AT CAMBRIDGE.

The University of Cambridge has erected new buildings for the use of students of anatomy; and it is hardly necessary to say that the authorities have made it their business to meet the requirements of the science of the times. Dr. Alexander Macalister, F.R.S., Professor of Anatomy at the University and Fellow of St. John's, gave an opening address of an historical character suitable for the occasion. It will probably surprise a good many people to find what a considerable number of anatomists and physiologists of some eminence have been associated with the University of Cambridge during the last three centuries. It will cause surprise, because, in spite of the undoubted fact, the University of Cambridge, has, until quite recently, behaved to medicine and the sciences auxiliary thereto as if she were ashamed of both. Anatomy, physiology, and medicine are still to some extent exotics both at Oxford and Cambridge. It is not that they are not studied there; they are, and a good deal of encouragement is given to their study by the more scientific members of both the universities. But what is unfortunate is that anatomy and physiology have taken no hold upon the general mind and life of the great body of young men who resort to the English universities. Cambridge is still a school of mathematics first, and of natural science a long way after. The best minds at Oxford are still devoted to classical learning.

It is not easy to advance arguments which will convince the leaders of thought and life at Oxford and Cambridge that the living study of human and comparative physiology would be like the discovery of a new intellectual world. Of its practical usefulness we do not now speak; but of its capacity for strengthening, expanding, correcting, and giving measure to intellectual activity there is no manner of doubt. The average educated man — we do not speak of the philosopher—is still a person extremely ignorant, full of the most stupid prejudices, mentally inelastic to a surprising degree, and, as a product of the higher education of the times, exceedingly disappointing. He has no just conception of the depths of his own ignorance or of the vast worlds of fact, and knowledge, and reason, and possibility, that lie beyond the range of his own studies. The average university man looks upon physiology as he looks upon needles and thread. It is a part of the doctor's professional equipment, exactly as the thread and the needles are parts of the tailor's armamentarium. But this is sheer barbarism; the outer darkness of the region of No-culture. The mere structure of the human body is infinitely marvellous; the functions associated with the structure are so wonderful as to be impossible of belief if they were not every-day phenomena. The mathematician, we submit, has nothing to offer half so wonderful, half so humbling, half so elevating to the mind. The classical scholar lives in a strange and fascinating past. He lives a life which, to some extent, disqualifies him for the life of the immediate present. If he devoted to the study of human and comparative physiology half the time he spends with the soldiers, the philosophers, the poets, the satirists, and the historians of Greece and Rome, he would not become a man of less power of understanding the men and women whose lives have such a fascination for him, but of more power. Greece and Rome would live for him as they never lived before; and, in addition, Britain and the British and all the wonderful world of to-day would also live before his eyes and be present to his touch.

Physiology is a breaker open of prison gates: it sets the mind free. It removes barriers and even mountains of tradition and custom that have no basis in right reason. It kills superstition; it makes the fool know he is a fool. It takes reasonable religion by the hand, and introduces her to all men as justice, reason, patience, hope, infinity. The man who has mastered the secrets, or some of the secrets, of the eye, the ear, the tongue, and the brain no longer excommunicates his fellow-man for holding a creed different from his own. On the contrary he holds his own creed with fear and trembling, except in so far as it is based upon a few of the leading principles of practical morals. Infallibility, he finds, is as impossible to a man's mind as is immortality to his present body.

Physiology is studied at Oxford and Cambridge as a mere matter of technicality. It is a part of the doctor's trade, as we have said, to so study it. That is pardonable at a hospital, which is a mere practical workshop and laboratory where the doctor learns his art; but a university exists for the study of man. Is there any student of man at the present time who is such a living anachronism as to believe that man can now be studied with any completeness apart from his physical organism? Does not even the most elementary teacher of mental science tell his students of reflexes as well as of intuitions; of molecular activity and of brain waste from brain friction in mental operations? But to the average University student all this is mere words, and evermore words. It always has been words, and nothing but words. But if the student knew the brain and the cord and the branches of the nervous system, and the miraculous functions which that system carries on, not only at its centre, but at its remotest circumference; not only in the operations of thought, and reasoning, and great purpose, and searchings into the infinite, but also in the perception of objects by the eye, by the ear, by the taste, by the touch; and, almost more wonderful still, the strange transformations it can make of the matter of the non-human world, and the power it has of building it up into its own organism, and even of recreating that organism in other similar forms—If he knew these things familiarly and intelligently he would find himself introduced to a world of miracles: of miracles which make the seeing and understanding man stand still in overwhelmed wonder. In their light his little prejudices, his supposed infallibilities, his ready anathemas would be seen to be the mere inconsequent triflings of a silly child, or the wild ravings of a lunatic.

Huxley and others have striven manfully, but to a large extent in vain, to persuade teachers to teach and learners to learn something about the wonders that are hidden in their own physical humanity. Those philosophers have pointed out that though by an accident physiology is a part of the doctor's art, yet in itself it is the only true and proper science of man. So far from its being second or third in a university curriculum of study, or, as it is sometimes, excluded altogether therefrom, it ought to be the very first of all the subjects of study, and other subjects should be secondary and auxiliary to it. The public elementary schools should put physiology first in the list of their work to be mastered; still more should the public schools and the universities teach us all to know ourselves in the only way in which we can be truly known. At present we are the slaves of the past, of Greece, of Rome, of Egypt, of Palestine. If we were physiologists the wisdom of the past would be our servant, as it was meant to be, and we should not be slaves at all. Ours would be the first free generation, and the parent of a true and glorious freedom for all generations yet to come.

The uses of physiology to the practical man have been purposely left out of the discussion. They demand not an article but a book to themselves. We have often asked in these pages what would be thought of the mere artisan who drives an engine if he knew nothing of the structure and use of the various parts of his engine? He would not be employed at all even in so humble a capacity as that of engine driver. But our greatest poets, statesmen, philosophers, and priests do all their thinking and speaking and working; they exert all the influence they can exert on their own and future generations altogether in virtue of the human body they possess and of its various parts; and yet not one of them either knows, or cares to know, anything at all about that body. Nay, these are the very classes of men who ban physiology, and insist that it shall be made a mere tool in the doctor's workshop. In the language of Carlyle, we live in a miraculous world; a world of stupidities incredible.

